# The Treatment of Pulmonary Emphysema in the Horse

**Published:** 1897-10

**Authors:** 


THE TREATMENT OF PULMONARY EMPHYSEMA IN THE HORSE. —Dr. Geaudeans reports his mode of treatment as being very efficacious, and he gives the following prescription: Arseniate of strychnia, 3 centigrammes; arseniate of iron, 25 centigrammes; iodide of potash, 2 grammes, to be administered three times daily. Frequent frictions of alcohol and vinegar generally produce a complete recovery in two months.—*Revue Vétérinaire.*

